# Host-pathogen-immune interactions in an air-liquid interface airway model

**DOI:** 10.3389/fcimb.2026.1788554

**Published:** 2026-04-10

**Authors:** Alexander F. Melanson, Annika Hettich, Claudia Antonella Colque, Jenny J. Persson, Pablo Laborda, Signe Lolle, Søren Molin, Helle Krogh Johansen

**Affiliations:** 1 Department of Clinical Microbiology 9301, Rigshospitalet, Copenhagen, Denmark; 2 Department of Experimental Medical Science, Section for Immunology, Lund University, Lund, Sweden; 3 The Novo Nordisk Foundation Center for Biosustainability, Technical University of Denmark, Lyngby, Denmark; 4 Department of Clinical Medicine, Faculty of Health and Medical Sciences, University of Copenhagen, Copenhagen, Denmark

**Keywords:** air-liquid interface (ALI), airway infection model, host-pathogen interaction, infection model, macrophages, *Pseudomonas aeruginosa*

## Abstract

**Background:**

Air–liquid interface (ALI) cell culture systems have improved the study of host-pathogen interactions in respiratory infections. However, most ALI models lack immune components, limiting their ability to capture epithelial–immune crosstalk. To address this, we developed a dual-cell ALI model incorporating human peripheral blood monocyte-derived macrophages beneath differentiated airway epithelial cells.

**Methodology:**

Macrophages were seeded on the basolateral side of transwell inserts using fibronectin coating. Model characterization included transepithelial electrical resistance (TEER) to assess epithelial barrier integrity, IL-8 secretion as a marker of epithelial inflammatory signaling, and confocal microscopy to evaluate cellular architecture before and after infection. Mono- and dual-cell cultures were infected with the laboratory strain *Pseudomonas aeruginosa* PAO1.

**Results:**

Macrophages adhered stably to the basolateral surface without compromising epithelial barrier integrity. Following infection, IL-8 secretion was elevated in epithelial monocultures compared to dual-cell cultures, suggesting early immune modulation in the presence of macrophages. While overall bacterial burden was comparable, confocal imaging revealed clustered bacterial growth in monocultures and a more dispersed spatial distribution in dual-cell cultures.

**Conclusions:**

This dual-cell ALI model enables investigation of early epithelial–immune interactions, inflammatory modulation, and bacterial colonization dynamics during airway infection. The system provides a versatile and human-relevant platform for studying respiratory host–pathogen interactions.

## Introduction

The rise of antibiotic-resistant bacteria has made persistent infections an increasingly serious threat to human health (Kavanagh, 2025; Mestrovic et al., 2025). There is therefore an urgent need to improve current infection models to accurately replicate the infected tissue environment for a more physiologically relevant understanding of the processes and mechanisms governing bacterial persistence. Traditional 2D cell culture systems have allowed the study and cultivation of individual human cell types, providing insights into the specific cellular responses to bacterial infection. Over the past 10–15 years, 3D cell culture model systems have been developed to bridge the gap between 2D culture and animal models (Baldassi et al., 2021; McCarron et al., 2021; Bauer et al., 2022; Moro et al., 2024). These systems enable researchers to better replicate the complex environments found in human tissues, thereby allowing studies of infections that closer align with physiological conditions. In the context of human airways, one commonly used model is the air-liquid interface (ALI) system, which utilizes a cell culture insert in conjunction with traditional culture dishes. These inserts provide an elevated culture surface, which supports the differentiation of airway epithelial cells into a pseudostratified epithelium, comprising basal, goblet, and ciliated cells (Colque et al., 2026). Moreover, the ALI model recapitulates key lung features, including mucus secretion, ciliary movement, and tight junction formation. In addition, primary cells can be derived from patient samples, allowing for a more personalized investigation strategy.

Previous studies using the ALI model to investigate infections with *Pseudomonas aeruginosa, Staphylococcus aureus*, and SARS-CoV-2 have examined immune responses, microbial colonization, epithelial barrier integrity, and molecular mechanisms underlying host-pathogen interactions (Baldassi et al., 2021; Ciszek-Lenda et al., 2023; La Rosa et al., 2025).

However, traditional ALI models usually lack immune cell components, limiting their utility to model immune interactions during infection. Given that immune cells are both resident and actively recruited, and bacteria need to evade their action to establish an infection, their inclusion is critical for obtaining a more realistic picture of host-pathogen interactions during infection. Macrophages were chosen for this model due to their pivotal role in the innate immune response as they are important pathogen-recognizing phagocytes constitutively present in tissues, and because they participate in both acute and chronic infections (Mitchell et al., 2016; Hill et al., 2017; Acosta and Alonzo, 2023; Ciszek-Lenda et al., 2023; Peña-Cearra et al., 2024; Sit et al., 2024). In mono-culture studies, macrophages infected with pathogens such as *Mycobacterium tuberculosis*, *Salmonella enterica*, and *P. aeruginosa*, have demonstrated altered intracellular signaling and spatial dynamics of bacterial colonization (Nix et al., 2007; Fallows et al., 2016; Maphasa et al., 2020; Moser et al., 2021; Ciszek-Lenda et al., 2023; Nickerson et al., 2024). Despite this, the complex interactions between airway epithelial cells, macrophages, and bacteria remain poorly understood.

Several studies have begun incorporating immune components into ALI-based airway models to better capture epithelial–immune interactions. For example, dendritic cells and macrophage-like cells have been introduced into ALI systems to investigate inflammatory signaling and pathogen response (Gras et al., 2017; De Rudder et al., 2020; Baldassi et al., 2021; Paplinska-Goryca et al., 2021). In many of these models, immune cells are added to the apical compartment or directly onto the epithelial surface, which facilitates immune–pathogen contact but can alter epithelial polarity and barrier organization. More recently, basolateral incorporation of macrophage-like cells has also been explored, including the use of THP-1–derived macrophages positioned beneath epithelial layers (De Rudder et al., 2020; Prescott et al., 2023; Burns et al., 2025). Despite these advances, there remains a need for models that maintain epithelial differentiation while allowing stable integration of primary human macrophages in physiologically relevant locations.

To address this, we developed a dual-cell ALI model that incorporates human peripheral blood monocyte-derived macrophages on the basolateral side of a differentiated airway epithelial layer. Macrophages were seeded to the basolateral side of cell culture inserts to mimic the homeostatic environment before infection. This model was infected with *P. aeruginosa*, a prevalent opportunistic pathogen able to cause persistent infections, especially in the airways of people with structural lung diseases (Wood et al., 2023).

This study aimed to (1) characterize macrophage adhesion to the cell culture insert, (2) assess the impact of macrophage presence on epithelial integrity, (3) quantify immune signaling via IL-8 secretion, and (4) investigate *P. aeruginosa* colonization, growth, and spatial organization in the presence and absence of macrophages.

## Materials and methods

### Preparation of cell culture inserts, BCi-NS1.1 cell line, and *Pseudomonas aeruginosa* PAO1

For the cell culture system, we used 1 μm pore polyester membrane inserts (Falcon). The BCi-NS1.1 cell line is an immortalized human airway basal epithelial cell line capable of differentiating into a mucociliary epithelium under ALI conditions (Walters et al., 2013; Prescott et al., 2023). Cells were generously obtained from Ronald G. Crystal (Weill Cornell Medical College, New York, USA). BCi-NS1.1 cell line was expanded and seeded into transwells and matured for 28 days as previously described (Laborda et al., 2024; Colque et al., 2026). Prior studies using BCi-NS1.1 ALI cultures have confirmed appropriate epithelial stratification, ciliation, and barrier formation through histological and transcriptomic characterization, supporting the structural integrity of this model (Walters et al., 2013; Laborda et al., 2024). To prepare the culture inserts for proper cell adhesion, type I collagen (Gibco) was added to the apical side of the inserts and incubated for 30 min at room temperature (RT). Subsequently, inserts were inverted and 20 µL fibronectin (Merck) at 5 µg/mL was added to the basolateral side and incubated for 2 h at 37 °C. For fibronectin coating optimization, two commonly used diluents were tested: (1) Dulbecco’s Modified Eagle Medium (DMEM, Gibco) and (2) Phosphate buffered saline (PBS, Gibco).

Preparation of the laboratory strain *P. aeruginosa* PAO1 inoculum and the infection procedure in the ALI model followed the protocol described by Laborda et al (Laborda et al., 2024). Two minor modifications were made: (1) the infecting inoculum was reduced from 1000 to 100 colony forming units (CFU), and (2) the infection duration was extended from 14 to 16 h. These adjustments were implemented to optimize infection dynamics for the dual-cell configuration and to allow sufficient time for monitoring epithelial–immune–bacterial interactions within the model. Immunostaining for epithelial differentiation markers (Muc5AC, p63, and CC10) was performed according to previously described protocols from our group (Laborda et al., 2024; Colque et al., 2026), including antibody sources, dilutions, and incubation conditions.

### Preparation of macrophages

Peripheral blood mononuclear cells (PBMCs) were isolated from fresh buffy coats obtained from healthy donors through the Department of Clinical Immunology, the Blood Bank, at Rigshospitalet, Copenhagen, Denmark. Buffy coats from 3–4 donors were pooled for each experiment to increase cell yield and minimize the impact of donor-specific variability. PBMCs were processed on the day of collection. In brief, 50 mL of buffy coat was diluted 1:1 with PBS supplemented with 2% fetal-bovine-serum (FBS, Gibco). PBMCs were isolated using 50 mL LEUCOSEP separation tubes (GREINER) pre-filled with Lymphoprep (STEMCELL Technologies). Following isolation, cells were washed twice in PBS + 2% FBS with 2 mM Ethylenediaminetetraacetic acid (EDTA) and centrifuged at 120 x g for 10 min at 4 °C to remove residual platelets. The resulting PBMCs were cryopreserved at a concentration of 1 x 10^7^ cells/mL in FBS supplemented with 10% dimethyl-sulfoxide (DMSO, Thermo Fisher Scientific).

Cryopreserved PBMCs were quickly thawed in a 37 °C water bath and immediately transferred into pre-warmed M0 base media consisting of RPMI 1640 (Gibco) supplemented with 10% FBS and 50 ng/mL macrophage colony-stimulating factor (M-CSF, Miltenyi Biotec). Monocytes were purified via negative selection using the MojoSort™ Human Pan Monocyte Isolation Kit (Biolegend), following the manufacturer’s instructions. Purified monocytes were seeded at a density of 14 x 10^6^ cells/mL in M0 base media in P-100 tissue culture-treated dishes and incubated at 37 °C with 5% CO_2_. Cells were differentiated into macrophages over 6 days, with a media change at 24 h to remove non-adherent cells.

For macrophage harvesting, TrypLE (Gibco) was applied to the culture plates and incubated at 37 °C for 5–10 min. Cells were lifted using a cell scraper and cold PBS was added to inactivate TrypLE and prevent macrophage adherence to collection tubes. Finally, cells were pelleted by centrifugation at 300 x g for 5–10 min and resuspended to the desired concentration for downstream processing and analysis.

### Establishing the dual-cell culture model

Airway epithelial cells, fully differentiated on the apical side of a transwell, were used to establish the dual-cell ALI model. Prior to macrophages seeding, TEER was measured to confirm epithelial integrity and media from the basolateral compartment was removed. To enable macrophage adhesion, culture inserts were inverted, and for the initial optimization fibronectin (Merck) was diluted in either DMEM or PBS. On day 0, fibronectin (5 µg/mL) was added to the basolateral side of the inserts and incubated for 2 h at 37 °C with 5% CO_2_. On day 27, fibronectin (5 µg/mL) was reapplied, and incubated for 30 min. Monocytes were differentiated separately into macrophages over six days before seeding and independently of BCi-NS1.1 epithelial cell differentiation. Subsequently, 20 µL of macrophage suspension (containing 8.5 x 10^4^ cells), was applied to the fibronectin-coated basolateral side and incubated for 1 h at 37 °C with 5% CO_2_. Control inserts without macrophages were treated identically, with macrophage-free media added to the basolateral side.

After incubation, media was aspirated and the inserts were carefully returned to their original orientation in the same 24-well plates. Each well contained 400 µL of pre-warmed media composed of a 1:1 mixture of M0 base media and Pneumacult-ALI maintenance media (STEMCELL Technologies), supplemented with 4 µg/mL heparin, 480 ng/mL hydrocortisone, Pneumacult-ALI 10x supplement, and Pneumacult-ALI maintenance supplement (all from STEMCELL Technologies). In control wells lacking epithelial cells, 50 µL of 1:1 mixture media was added apically to maintain comparable conditions. All cultures were incubated at 37 °C in a humidified 5% CO_2_ incubator until the time of infection.

### Immunostaining and confocal microscopy of dual-cell culture model

Before staining, both the apical and basolateral compartments of the transwells were rinsed once with PBS. Cells were then fixed by adding 4% paraformaldehyde (PFA, Thermo Fisher Scientific) to the apical (200 µL) and basolateral (400 µL) compartments and incubated for 20 min at 4 °C. After fixation, PFA was removed, and samples were washed twice with PBS. Fixed membranes were excised from the culture inserts with a surgical scalpel and placed on a parafilm-covered rectangular petri dish. Membranes were then blocked with 100 µL of 3% goat serum (Jackson Immuno) in PBS for 1 h at RT. Droplet-based staining ensured that both the apical and the basolateral surfaces remained in contact with solution, thereby enhancing visualization of macrophages attached to the basolateral side. Primary antibodies were diluted in blocking buffer and applied to membranes in 100 µL droplets. Membranes were incubated overnight at 4 °C with rabbit anti-CD14 (Thermo Fisher Scientific) and acetylated alpha-tubulin (Santacruz Biotechnology) antibodies. Following incubation, membranes were washed three times with PBS. Secondary staining was performed using goat anti-rabbit antibody (Thermo Fisher Scientific) diluted in blocking buffer, applied in 100 µL droplets, and incubated for 1 h at RT. For intracellular staining, cells were permeabilized using 1% saponin (Sigma Aldrich) in blocking buffer applied as 100 µL droplets for 30 min at RT. Nuclei were counterstained with DAPI (4′, 6-diamidino-2-phenylindole). Different nuclear stains were used depending on the imaging setup and fluorophore compatibility. To-Pro-3 was used in some confocal imaging experiments to optimize spectral separation between epithelial, macrophage, and bacterial signals for 10 min at RT, followed by three PBS washes, prior to antibody application. Z-stack images were acquired using a step size of 0.1 µm. The same imaging settings and processing parameters were applied to all conditions.

For mounting, membranes were placed on glass slides, covered with a drop of VECTASHIELD^®^ Antifade Mounting media (VWR), and sealed with a coverslip using nail polish. Confocal images were acquired using a Leica Stellaris 8 confocal microscope (40x oil immersion objective, NA 1.3).

### Flow cytometry and staining of airway epithelial cells

Basal cell differentiation was assessed after 28 days of ALI culture. To remove accumulated mucus, the apical side of each transwell was treated with 200 µL of PBS containing 10 mM dithiothreitol (DTT, Sigma Aldrich) and incubated for 10 min at 37 °C in a 5% CO_2_ humidified incubator. Residual DTT was removed by three washes with 75 µL of PBS. The media from the basolateral compartment was aspirated and enzymatic dissociation of the cell layer was performed using trypsin (Gibco) supplemented with 0, 5 mM EDTA. A volume of 500 µL was added to the basolateral compartment and 200 µL to the apical compartment. Cells were incubated for 10–20 min at 37 °C in a 5% CO_2_ humidified incubator. Apical compartment cells were harvested by pipetting and transferred to a 1.5 mL Eppendorf tube. The basolateral trypsin solution was then used to rinse the insert twice to maximize cell recovery. To stop trypsin activity, 500 µL of complete media (RPMI 1640 with 20% FBS) was added to each tube. The cell suspensions were filtered through a 100 µm filter to reduce clumping and collected in a 15 mL Falcon tube containing 3 mL of complete media. The filter was then rinsed with 1 mL of complete media to ensure maximal cell recovery. Cells were centrifuged at 400 x g for 5 min at RT. The supernatant was removed, and the cell pellet resuspended in 1 mL of 0.5% (v/v) PFA diluted in PBS, transferred to a clean 1.5 mL Eppendorf tube, and incubated for 8 min at RT. Cells were subsequently centrifuged at 300 x g for 5 min and washed twice with PBS to remove residual PFA. Fixed cells were either used immediately for flow cytometry analysis or stored at -80 °C for later use.

For staining, cells were resuspended in 100 µL staining buffer consisting of 5% (v/v) goat serum (Jackson Immuno) in PBS and incubated for 15 min at RT on a rotator (350 x g) to block non-specific primary antibody binding, including Fc receptor interactions. Antibodies against the surface proteins CD271 and CD66c (Thermo Fischer Scientific) were added directly to the blocked cells and incubated for 30 min at RT on a rotator (350 x g), protected from light. After surface staining, cells were washed three times with 700 µL of PBS. For intracellular staining, cells were permeabilized in a 100 µL staining buffer containing 0.2% (w/v) saponin (Sigma Aldrich) for 15 min at RT on a rotator (350 x g). Antibodies targeting intracellular markers Muc5AC and TUBA (Thermo Fisher Scientific) were then added directly to the permeabilized cells and incubated for 30 min at RT on a rotator (350 x g), protected from light.

After staining, cells were washed three times in 700 µL PBS supplemented with 0.1% saponin and resuspended in 250 µL PBS containing 0.1% bovine serum albumin (Sigma-Aldrich). The stained cells were transferred to a flat-bottom 96-well plate (Greiner). Samples were analyzed using an Agilent Novocyte flow cytometer. To correct for fluorescence spillover, spectral overlap compensation was performed using the Mouse Ig, κ/Negative Control Compensation Particles Set (BD Biosciences), with each antibody used in the experiment. Unstained cells were included as reference.

### Flow cytometry analysis of macrophages

Harvested macrophages were stained with fixable viability dye eFluor 450 (Thermo Fisher Scientific), followed by fixation in 4.2% PFA (BD Bioscience), for 15 minutes at RT. Non-specific Fc receptor interactions were blocked using human Fc blocking reagent (BD Bioscience) for 30 min at RT, and cells were stained with anti-CD16-PE and anti-CD14-FITC (Thermo Fisher Scientific). Samples were analzyed using a NovoCyte Quanteon flow cytometer (Agilent) using ultracomp ebeads™ (Thermo Fisher Scientific) for compensations, as well as fluorescent minus one (FMO) were also used as a compensation control.

### Quantification of infection parameters after *P. aeruginosa* infection

To determine bacterial distribution across the model, three fractions were collected. The apical fraction was obtained by washing the apical compartment PBS and represents planktonic and loosely associated bacteria at the epithelial surface. Following apical washing, epithelial layers were treated with Triton X-100 to recover the epithelium-associated bacterial fraction, which includes bacteria tightly bound to the epithelial surface as well as bacteria located within or between epithelial cells. The basolateral fraction was collected from the basolateral medium and represents bacteria that traversed the epithelial barrier into the lower compartment.

All samples were serially diluted and plated in triplicate on Luria–Bertani (LB) agar plates for CFU quantification (CFU/mL). In addition, Transepithelial electrical resistance (TEER) was measured using a voltohmmeter (e.g., EVOM2, World Precision Instruments) with STX electrodes according to the manufacturer’s instructions. Measurements were performed at room temperature, and values were corrected by subtracting blank insert resistance and multiplying by the membrane surface area. Interleukin-8 (IL-8) secretion was assessed following infection. IL-8 concentrations were measured using an enzyme-linked immunosorbent assay (ELISA). All procedures were performed as previously described (Laborda et al., 2024).

### Data analysis

Flow cytometry data was analyzed using the Flow Cytometry Analysis Software Flowlogic (Version 7.3), and doublet discrimination was performed using forward scatter area versus height (FSC-A/FSC-H) gating to exclude cell aggregates before downstream analysis (**Supplementary Figures 1A, B**, for the gating strategy). Confocal images were analyzed and edited using the Fiji (software based on Windows Image J version 2.14.0/1.54f). Z-stack images obtained by confocal microscopy were Z-projected using the maximum intensity projection. All plots and statistics were generated with GraphPad Prism Version 10.5.0 for Mac and the Brown–Forsythe test prior to statistical analysis (GraphPad Software, San Diego, California USA). Data is presented as mean ± SD from three separate biological replicates, each with three technical replicates. Statistical significance was determined by t-test or one-way ANOVA with Tukey’s multiple comparisons test and is indicated as *p ≤ 0.05, **p ≤ 0.01, ***p ≤ 0.001 and ****p < 0.0001.

## Results

### Macrophage adhesion to cell culture inserts

**Figure 1:**
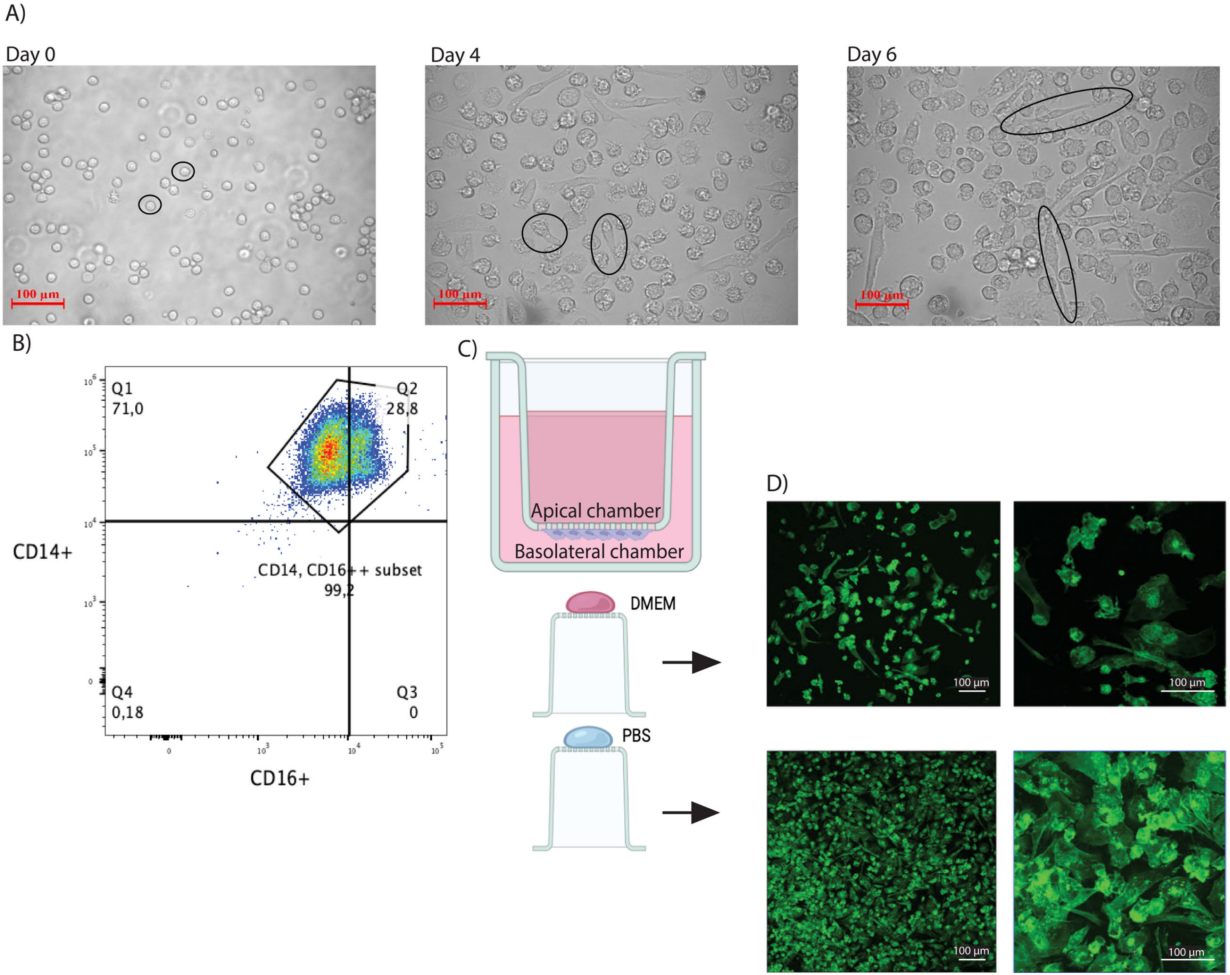
Maturation and phenotype development of human peripheral blood monocyte-derived macrophages and adhesion to air-liquid-interface cell culture inserts. **(A)** Development of monocytes into macrophage-like cells during *in vitro* culture. Light microscopy images (magnification: 20x) of cellular phenotypes at day 0 (after negative selection), day 4, and day 6. **(B)** Flow cytometry-based characterization of macrophages. **(C)** Schematic cartoon of macrophage seeding to the basolateral side of the cell culture inserts, image was created in BioRender *by Alexander F. Melanson* (2025) https://BioRender.com/lwgs8nj. **(D)** Adherence of macrophages to cell culture inserts coated with fibronectin diluted in Dublecco’s Modified Eagle Medium (DMEM, top) or Phosphate Buffered Saline (PBS, bottom). Confocal microscopy images of macrophages stained with anti-CD14 (green). Data are representative of three biological replicates, each with three technical replicates.

The primary objective of this study was to establish a dual-cell culture model incorporating monocyte-derived macrophages to an airway epithelial cell culture system. To achieve this, macrophage adhesion to culture inserts was first evaluated. Human monocytes were differentiated into macrophages over six days in the presence of macrophage colony-stimulating factor (M-CSF). Brightfield microscopy confirmed the expected elongated morphology of adherent macrophages (**Figure 1A**). Surface marker analysis by flow cytometry showed robust expression of CD14 and CD16, with similar patterns of marker expression as previously described (Buckner et al., 2011; Lee et al., 2020), confirming successful macrophage differentiation (**Figure 1B**).

Macrophages were seeded onto the basolateral side of cell culture inserts (**Figure 1C**) coated with human plasma fibronectin diluted in either DMEM or PBS to identify the most efficient fibronectin coating diluent (**Figure 1D**). Confocal microscopy of CD14-stained macrophages showed cellular adhesion in both conditions, although visual assessment suggested fibronectin diluted in PBS better supported this feature (**Figure 1D)**.

### Establishment of dual-cell culture model

**Figure 2:**
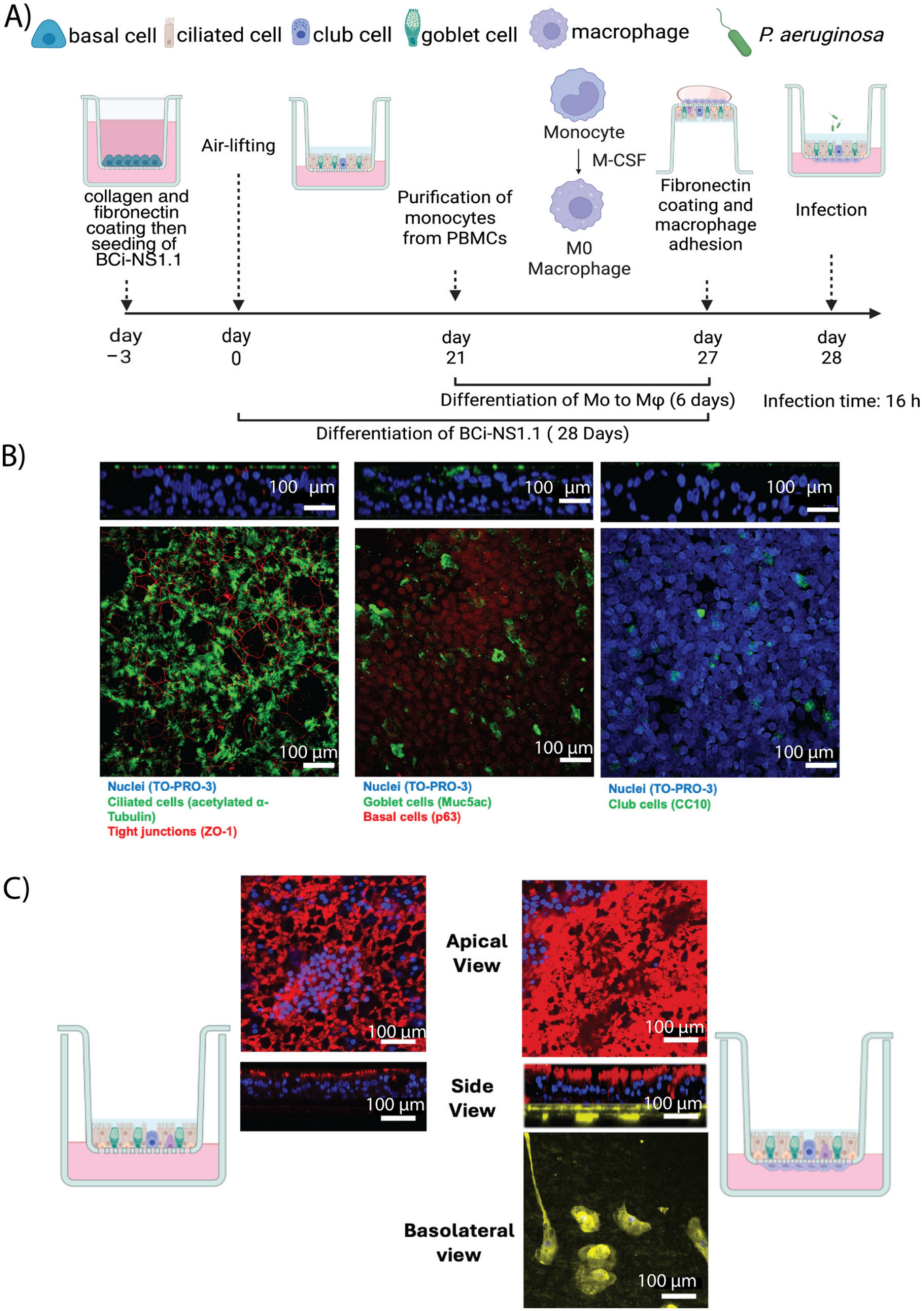
Establishment of the dual-cell culture infection model. **(A)** Schematic timeline of the process to establish the model image was created in BioRender by *Alexander F. Melanson* (2025) https://BioRender.com/lwgs8nj. **(B)** Confocal images of BCi-NS1.1 cells: left panel shows staining of cell nuclei (blue, To-pro-3), ciliated cells (green, acetylated alpha-tubulin), and tight junctions (red, ZO-1); middle image, nuclei (blue, To-pro-3), goblet cells (green, Muc5ac), and basal cells (red, p63); right panel, nuclei (blue, To-pro-3) and club cells (green, CC10). **(C)** Left, first a schematic cartoon of a cell culture insert system without macrophages, then a mono-cell culture insert with only BCi-NS1.1 cells with ciliated cells (red, acetylated alpha-tubulin), and nuclei (blue, DAPI). Right, dual-cell culture model, with macrophages (yellow, CD14) adhering to the basolateral side of the cell culture insert. Then another schematic cartoon, this one showing macrophages adhered to the basolateral side of transwells. Apical view for both left and right images are a maximum projection of the Z-stacks.

The sequential steps from ALI model differentiation, macrophage adhesion, to dual-cell culture establishment is displayed in **Figure 2A**. Fully differentiated BCi-NS1.1 airway epithelial cells were used to generate the dual-cell model. Differentiated macrophages were subsequently seeded onto the basolateral side of the epithelial cultures prior to infection, enabling establishment of the dual-cell ALI system for downstream analyses.

To assess the cellular composition of the epithelial layer, transwell inserts containing fully differentiated airway epithelial BCi-NS1.1 cells were stained and analyzed by confocal microscopy (**Figure 2B**). Differentiation led to a fully mature pseudostratified epithelial tissue, characterized by the formation of tight junctions and the presence of multiple cell types representative of an airway epithelium (**Supplementary Figures 2A, B**).

**Figure 2C** shows the mono-culture model (epithelial tissue only) and the dual-cell culture model (epithelial tissue and macrophages). Because of the relative instability of fibronectin, the wells were supplemented with fresh fibronectin prior to macrophage seeding to ensure their adhesion, which was confirmed by confocal imaging (**Figure 2D**). To evaluate the retention of adherent cells, detached macrophages were collected and counted 24 h after seeding them. No significant loss of macrophages was observed (approximately 0.006% to 0, 009% macrophages detached).

Importantly, the addition of macrophages had no measurable effect on epithelial barrier function, as TEER values remained unchanged (**Supplementary Figure 2C**). These findings confirm that macrophages can be successfully incorporated into the basolateral side of the culture insert beneath a fully differentiated airway epithelium without compromising epithelial integrity.

### Infection of mono- and dual-cell culture model

**Figure 3:**
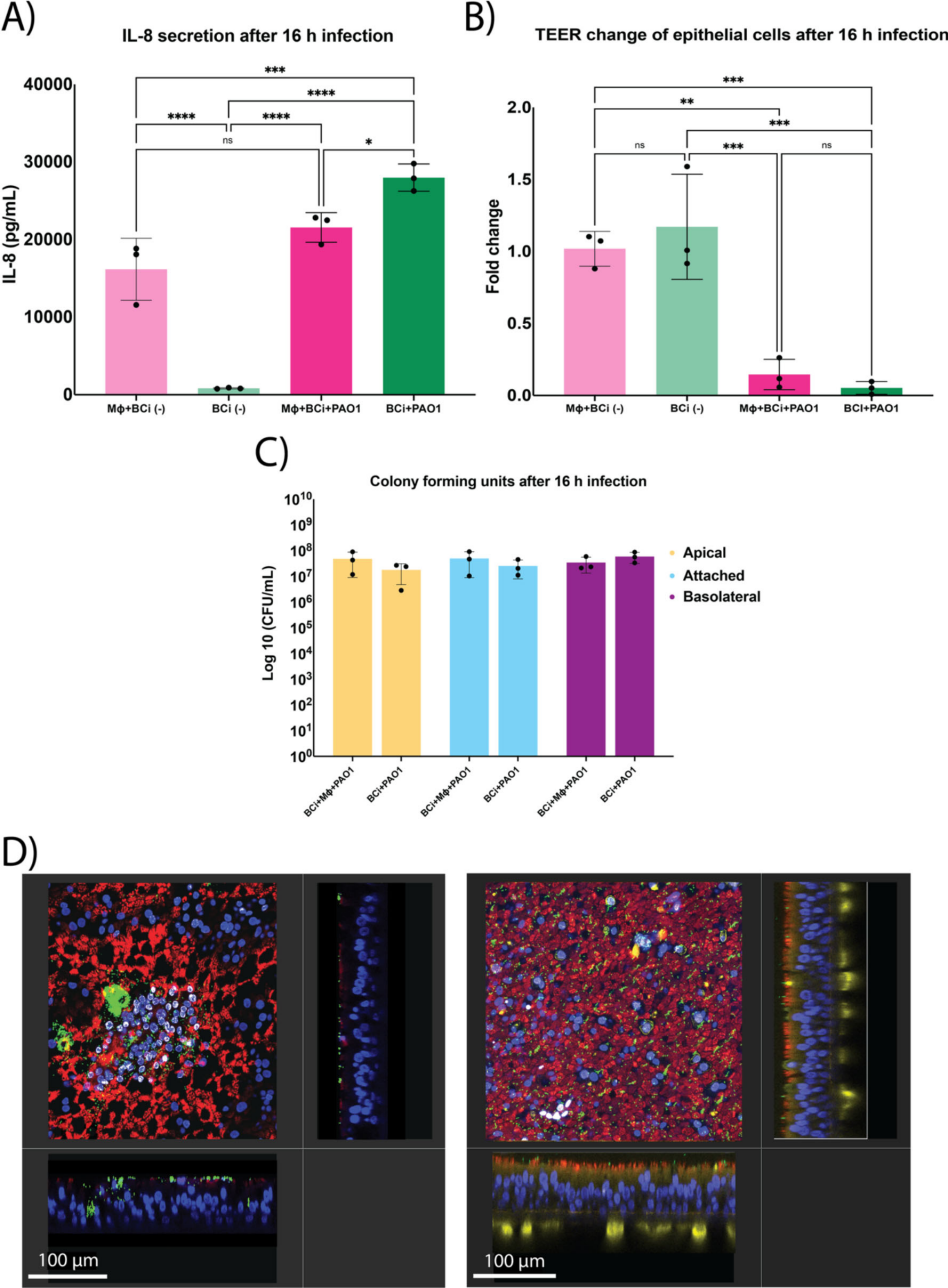
Effect of Pseudomonas aeruginosa infection in mono- and dual-cell culture models. **(A)** IL-8 quantification (pg/mL) after 16 h of infection with (P) aeruginosa. **(B)** Fold change of Transepithelial Electrical Resistance (TEER) (Ω·cm^2^) in both models after infection with (P) aeruginosa for 16 h. **(C)** Bacterial load in colony-forming units (CFU/mL) across the different compartments of the dual-cell air-liquid-interface model, apical, attached (bacteria present within the epithelial cells or firmly attached to the surface), and the basolateral compartment. **(D)** Orthogonal and cross-sectional views of the mono- and dual-cell culture model after 16 h of infection with (P) aeruginosa (scale bar: 100 µm). Left image, a mono-cell culture infection model showing ciliated cells (red, acetylated alpha-tubulin), (P) aeruginosa (green, sfGFP), and cell nuclei (blue, DAPI); right image shows a dual-cell culture model with ciliated cells (red, acetylated alpha-tubulin), (P) aeruginosa (green, sfGFP), cell nuclei (blue, DAPI), and macrophages (yellow, CD14). All Images were taken at 40X magnification. Data represent mean ± SD from three biological replicates, each with three technical replicates. Statistical significance was determined by one-way ANOVA with Tukey**’**s multiple comparisons test for IL-8, TEER and CFU comparisons, and is indicated as *p ≤ 0.05, **p ≤ 0.001, *** p ≤ 0.0001, and ****p < 0.0001.

Both mono- and dual-cell culture models were infected with 100 CFU of the laboratory strain *P. aeruginosa* PAO1 and incubated for 16 h. To test differences in epithelial immune response between the two models, IL-8 secretion was measured as a marker of ability for immune cell recruitment (**Figure 3A**). Under mock conditions, IL-8 secretion was significantly higher in the dual-cell model compared with the epithelial monoculture (one-way ANOVA with Tukey’s multiple comparisons test, p < 0.0001). Upon PAO1 infection, IL-8 levels increased significantly in the epithelial monoculture relative to mock controls (one-way ANOVA with Tukey’s multiple comparisons test, p < 0.05). In contrast, PAO1 infection did not result in a significant increase in IL-8 secretion in the dual-cell culture model. Consequently, IL-8 levels in infected epithelial monocultures were significantly higher than in infected dual-cell cultures (one-way ANOVA with Tukey’s multiple comparisons test, p < 0.05).

To investigate the effect of infection on the epithelial cell layer integrity, TEER was measured post-infection (**Figure 3B**). Under mock conditions, no significant difference in TEER was observed between epithelial monocultures and dual-cell cultures containing macrophages.

Infection with *P. aeruginosa* PAO1 caused a significant reduction in TEER compared with corresponding mock controls in both mono- and dual-cell models (one-way ANOVA with Tukey’s multiple comparisons test, p ≤ 0.0001). TEER values in infected epithelial monocultures were also significantly lower than those in mock dual-cell cultures (p ≤ 0.001). Importantly, no significant difference in TEER was detected between infected mono- and dual-cell models, indicating that macrophage presence does not prevent the initial epithelial barrier disruption induced by *P. aeruginosa*.

Bacterial burden in the apical, attached, and basolateral compartments, revealed comparable bacterial loads across models (**Figure 3C**, one-way ANOVA with Tukey’s multiple comparisons test, p>0.05). Visualization of the cell culture inserts showed (**Figure 3D**) large bacterial aggregates consistent with the known aggregate-forming behavior of *P. aeruginosa* (Rossi et al., 2021; Laborda et al., 2024; Leoni Swart et al., 2024). In contrast, in the dual-cell culture model, a more dispersed bacterial distribution with predominantly single bacteria present throughout the epithelium was observed.

## Discussion

This study introduced a dual-cell ALI airway model containing epithelial cells and monocyte-derived macrophages. The aim was to establish a more realistic airway infection model system. Previous ALI models often only relied on epithelial monocultures or limited immune additions (Gras et al., 2017; Baldassi et al., 2021; Paplinska-Goryca et al., 2021). Here, we successfully adhered macrophages into basolateral membrane of the ALI model while preserving epithelial barrier integrity and maintaining macrophage phenotype, as indicated by typical morphology and sustained CD14 expression (Hamidzadeh et al., 2020; Paplinska-Goryca et al., 2021; Hickman et al., 2023). This dual-cell configuration supports the study of interactions between epithelial and immune cells and enables simultaneous analysis of microbial behavior and early host responses. Recent studies have begun integrating immune cells into ALI airway models, including the incorporation of dendritic cells and macrophage-like cells in either apical or basolateral configurations (Gras et al., 2017; De Rudder et al., 2020; Baldassi et al., 2021; Paplinska-Goryca et al., 2021; Prescott et al., 2023; Burns et al., 2025). While these systems represent important advances, many rely on immortalized cell lines or position immune cells directly on the apical surface, which may disrupt epithelial organization. In contrast, our model uses primary human monocyte-derived macrophages seeded on the basolateral side of a fully differentiated epithelium. This configuration preserves epithelial architecture while enabling immune–epithelial communication and therefore provides a complementary platform for studying early host–pathogen interactions.

Macrophages were included because they serve as key immune sentinels in the airways. Under homeostatic conditions, monocytes enter from the bloodstream and differentiate beneath the epithelium. From this position, macrophages survey the airways by extending protrusions through tight junctions and migrate into the airway lumen when infection occurs (Meeker et al., 2012; Chikina et al., 2020; Pérez-Rodríguez et al., 2022; Yang et al., 2023). To reflect this anatomy, we seeded macrophages on the basolateral membrane building on previous studies that have positioned other myeloid-derived immune cells such as dendritic cells and macrophages directly in the apical chamber (Gras et al., 2017; Baldassi et al., 2021; Paplinska-Goryca et al., 2021; Misiukiewicz-Stępien et al., 2022). As a result, our model retains the natural spatial organization of airway tissue and immune cells.

Technical optimization was essential for the dual-cell culture system, and membrane pore size was one of the key parameters. Previous work has shown that very small pores (≤0.4 µm) can restrict macrophage migration and limit transepithelial probing because they prevent cells from extending their projections across the membrane (Pérez-Rodríguez et al., 2022; Yang et al., 2023; Li et al., 2025). In contrast, large pores such as 4 µm can weaken epithelial barrier formation. Based on these observations, we selected a 1.0 µm pore size to balance both requirements. Together, these considerations highlight how membrane properties directly influence immune–epithelial communication in ALI-based co-culture models.

Macrophage adhesion also required refinement. A single fibronectin coating, applied according to standard instructions, was insufficient. After testing, we added a second short incubation period of 30 min with fibronectin immediately before seeding. This step improved macrophage attachment without removing epithelial media long enough to compromise epithelial integrity. With this adjustment, macrophages remained stably adhered throughout infection experiments.

*P. aeruginosa* organization on the epithelial surface changed noticeably in the presence of macrophages. Consistent with previous reports, epithelial monocultures supported the formation of bacterial clusters (Moser et al., 2021; Ciofu et al., 2022; Laborda et al., 2024; Leoni Swart et al., 2024). However, introducing macrophages resulted in a more dispersed distribution, suggesting that early macrophage activity alters the spatial dynamics of bacterial attachment. Interactions between macrophages and *P. aeruginosa* are known to influence early infection dynamics, including modulation of cytokine signaling, phagocytic responses, and bacterial spatial organization within airway environments (Moser et al., 2021; Ciszek-Lenda et al., 2023). Although functional macrophage responses were not the primary focus of this methodological study, the observed changes in bacterial distribution suggest that incorporating macrophages into ALI systems may reveal early immune-mediated effects that are not captured in epithelial monoculture models. Although our observations were qualitative, they indicate a possible change in bacterial infection phenotype. This shift in spatial organization may represent an early macrophage-mediated containment mechanism, occurring before overt phagocytosis or bacterial clearance. However, confocal images are presented as representative projections and may not always reflect the exact quantitative distribution of bacteria across compartments. Quantitative spatial analysis in future work will help determine whether macrophage presence actively disrupts microcolony initiation or merely prevents their maturation.

The reduction in IL-8 when macrophages were present suggests that macrophages shape epithelial inflammatory output during the earliest stages of infection. This interpretation aligns with work showing that macrophages can attenuate epithelial chemokine release to prevent excessive neutrophil recruitment (Bishayi et al., 2017; Moser et al., 2021; Toya et al., 2024). Similar attenuation of pro-inflammatory cytokine release, including IL-8, has been observed in direct epithelial–macrophage co-culture airway models exposed to environmental stressors, further supporting a regulatory role for macrophage–epithelial crosstalk in shaping epithelial inflammatory output (Burns et al., 2025). Under mock conditions, macrophage inclusion led to increased IL-8 secretion, which may reflect basal immune–epithelial communication rather than a pathological inflammatory response. Complementing this, TEER measurements confirmed that baseline epithelial barrier integrity was preserved in the dual-cell model, indicating that macrophages do not disrupt epithelial structure under homeostatic conditions. Together, these findings indicate that while macrophages modulate epithelial inflammatory signaling, they do not lead to initial epithelial barrier loss. Our results highlight that epithelial responses measured in monoculture may overestimate *in vivo* inflammation and underscore the importance of incorporating immune cells to capture early host-pathogen interactions. Distinguishing whether IL-8 modulation occurs through receptor-mediated sequestration, altered transcription, or contact-dependent signaling remains an important future direction.

Our model provides several advantages over standard ALI systems. It maintains physical separation between cell types while enabling direct communication. It can also be adapted to other pathogens or immune cell types. Together, these features make it suitable for studying epithelial-immune cell interactions during bacterial, viral, and fungal infections.

However, this system has some limitations. The static transwell design does not reproduce airflow, mechanical stretch, or mucus transport and longer infection periods would also have allowed us to study in-depth biofilm maturation. The macrophages that we added to the model system were blood-derived rather than fully tissue-resident. In addition, incorporation of patient-derived cells could further have supported personalized infection research.

Despite these limitations that can be addressed in future work, this dual-cell ALI model offers a realistic and controllable platform for airway infection studies. It bridges the gap between monocultures and *in vivo* systems and provides a useful tool for investigating respiratory immune responses and evaluating targeted therapeutic strategies.

## Data Availability

The raw data supporting the conclusions of this article will be made available by the authors, without undue reservation.
