# Supplementary material for: Host-pathogen-immune interactions in an air-liquid interface airway model
Source: Front Cell Infect Microbiol. 2026 Apr 10;16:1788554. doi: 10.3389/fcimb.2026.1788554 (PMC13106080; doi:10.3389/fcimb.2026.1788554)
Supplement: Supplementary file 1 [file Image1.pdf]

Supplementary Figure 1

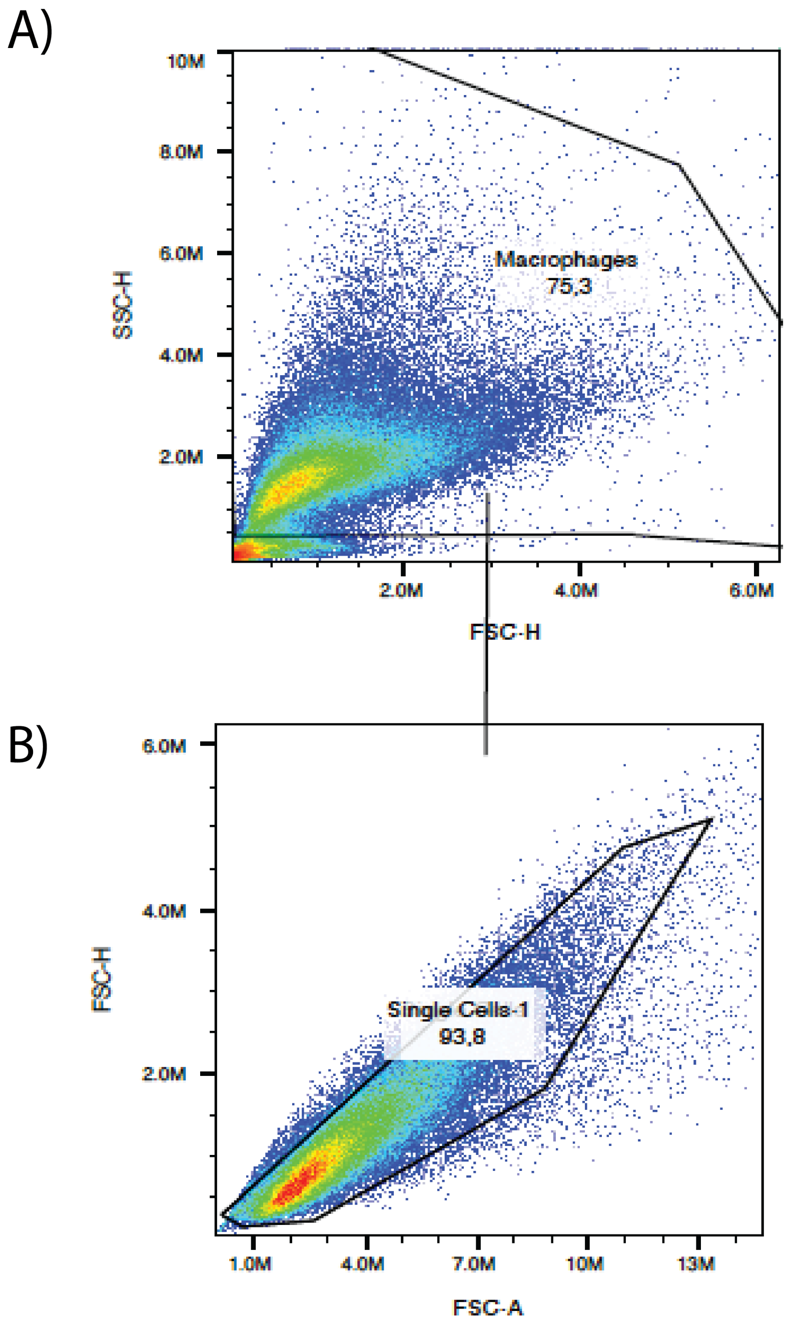

Supplementary Figure 1. Flow cytometry gating strategy for identification of single-cell populations. A) Flow cytometry gating strategy used to select single-cell macrophage populations. Debris was excluded based on forward and side scatter parameters. B) Doublet discrimination was then performed using forward scatter area versus forward scatter height

(FSC-A/FSC-H) gating to remove cell aggregates and ensure that only single cells were included in downstream analysis.
