# Supplementary material for: Host-pathogen-immune interactions in an air-liquid interface airway model
Source: Front Cell Infect Microbiol. 2026 Apr 10;16:1788554. doi: 10.3389/fcimb.2026.1788554 (PMC13106080; doi:10.3389/fcimb.2026.1788554)
Supplement: Supplementary file 2 [file Image2.pdf]

Supplementary Figure 2

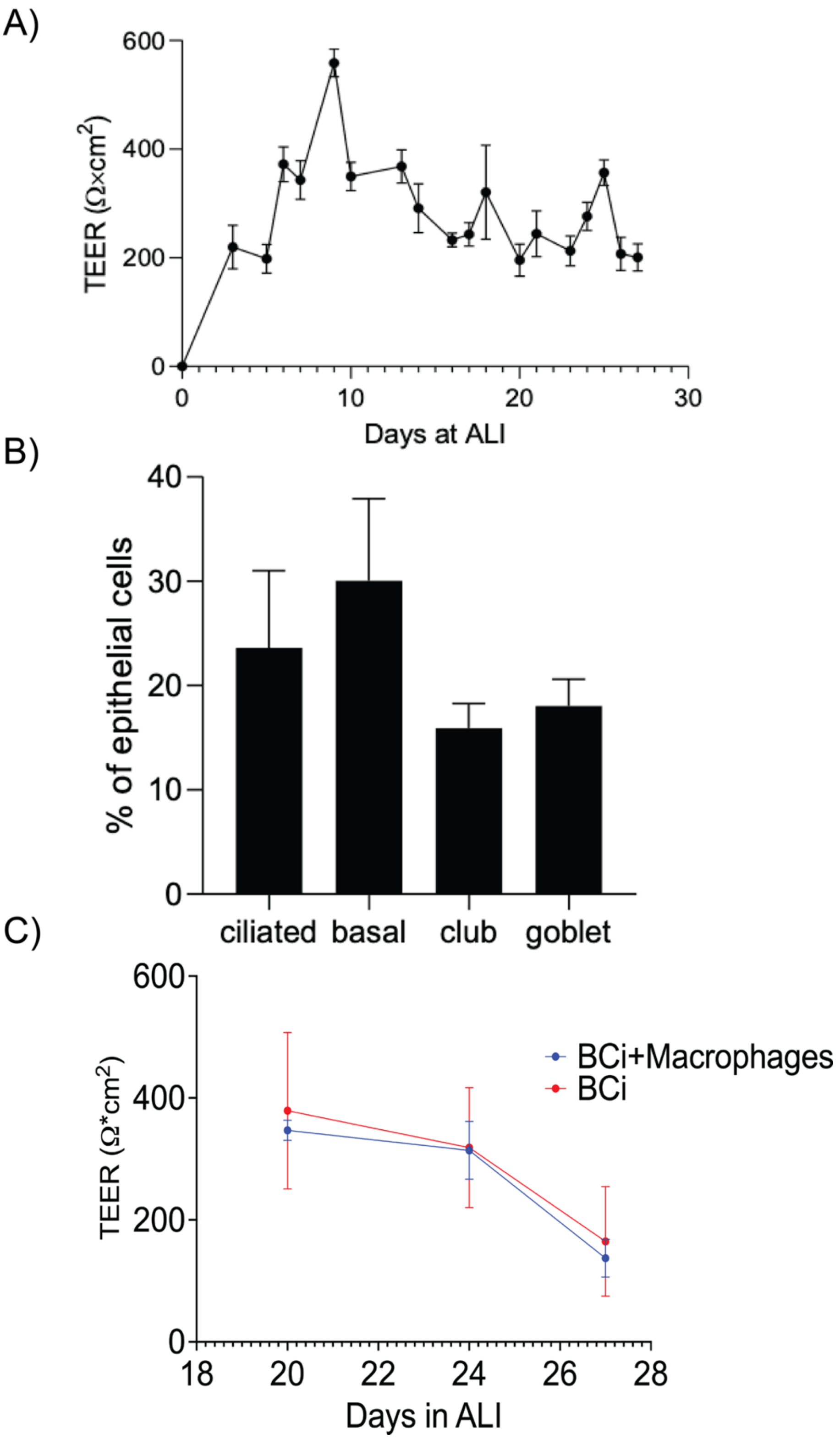

Supplementary Figure 2. Epithelial barrier and cell population development, and surface integrity after macrophage adhesion. A) Changes in Trans-Epithelial-Electrical Resistance (TEER) values as the BCi-NS1.1 cells mature over 28 days. B) Percentage of cells present in the mono-cell culture model were analyzed using flow cytometry. C) TEER values of mono- and dual-cell models before and after addition of macrophages (red: BCi-NS1.1 cells alone, blue: BCi-NS1.1+Macrophages). Data represent mean ± SD from three biological replicates, each with three technical replicates.
